# Mitral Annular Disjunction Assessed Using Cardiac MR Imaging in Pediatric Patients

**DOI:** 10.3390/diagnostics15222857

**Published:** 2025-11-12

**Authors:** Şükriye Yılmaz, Berna Ucan, Hasan Bulut, Senem Özgür, Tamer Yoldaş, Pelin Altınbezer

**Affiliations:** 1 Department of Pediatric Radiology, Ankara Etlik City Hospital, Ankara 06170, Turkey; dryavuzer@gmail.com (Ş.Y.); hsn_blt89@hotmail.com (H.B.); 2 Department of Pediatric Cardiology, Ankara Etlik City Hospital, Ankara 06170, Turkey

**Keywords:** mitral annular disjunction, mitral valve prolapse, pediatric cardiac magnetic resonance imaging

## Abstract

**Background/Objectives**: Mitral annular disorders constitute a heterogeneous group of structural abnormalities that can significantly influence morbidity and mortality in both adult and pediatric populations. Advances in cardiac magnetic resonance (CMR) imaging have refined the ability to characterize these disorders with high spatial resolution and reproducibility. Among them, mitral annular disjunction (MAD) and mitral valve prolapse (MVP) have emerged as interrelated entities implicated in valvular dysfunction, arrhythmogenesis, and myocardial remodeling. This study aimed to determine the prevalence of MAD in a pediatric cohort, explore its association with MVP, and delineate related CMR findings, including myocardial fibrosis. **Methods**: A retrospective review was conducted in 295 pediatric patients who underwent clinically indicated CMR between September 2022 and June 2025. Echocardiographic and CMR data were systematically compared for the detection of MAD, MVP, and mitral regurgitation (MR). MAD length and mitral annular measurements were obtained from two-chamber and left ventricular outflow tract (LVOT) cine sequences. Late gadolinium enhancement (LGE) was evaluated to identify myocardial fibrosis. **Results**: MAD was detected more frequently by means of CMR than echocardiography (23.2% vs. 9.3%), as was MVP (34.2% vs. 22.4%), whereas MR was more often observed on echocardiography (31.2% vs. 15.2%). Inter-modality agreement was moderate for MAD, moderate-to-substantial for MVP, and fair for MR. LGE was identified only in patients with concomitant MAD and MVP, suggesting limited myocardial involvement in isolated MAD. **Conclusions**: CMR demonstrates superior sensitivity in detecting MAD and MVP compared with echocardiography and allows for early recognition of systolic–diastolic annular dissociation before advanced myocardial remodeling occurs. These findings underscore the clinical utility of CMR as a complementary modality for comprehensive assessment, risk stratification, and follow-up of pediatric patients with suspected mitral annular abnormalities.

## 1. Introduction

Mitral annular diseases represent an important subset of valvular pathologies contributing to cardiovascular morbidity and mortality across all age groups, including pediatric populations [1]. The diagnostic yield of these disorders has increased considerably with the evolution of high-resolution imaging technologies and the growing expertise of specialized cardiovascular centers [2]. Early and accurate identification of structural valve abnormalities is critical, as it provides insights into underlying pathophysiology and facilitates timely intervention in children presenting with cardiogenic symptoms.

**Figure 1:**
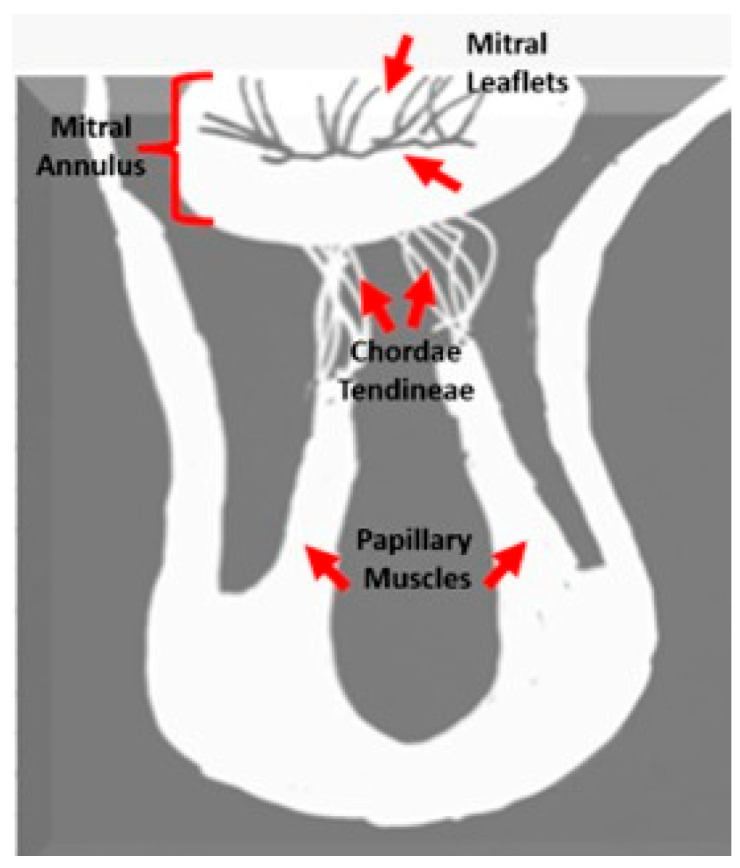
Mitral valve anatomy.

Anatomically, the mitral valve apparatus is a dynamic, multi-component structure comprising the anterior and posterior leaflets, the fibrous mitral annulus, and the subvalvular apparatus—consisting of the papillary muscles and chordae tendineae. The leaflets are anchored to the annulus encircling the atrioventricular junction, while the chordae provide symmetrical tethering to maintain valve competency throughout the cardiac cycle. Alterations in the geometry or integrity of any of these components can result in mitral valve dysfunction, ranging from benign prolapse to complex regurgitant lesions [3]. Figure 1 illustrates the anatomical components of the mitral valve apparatus, including the mitral annulus, leaflets, chordae tendineae, and papillary muscles.

**Figure 2:**
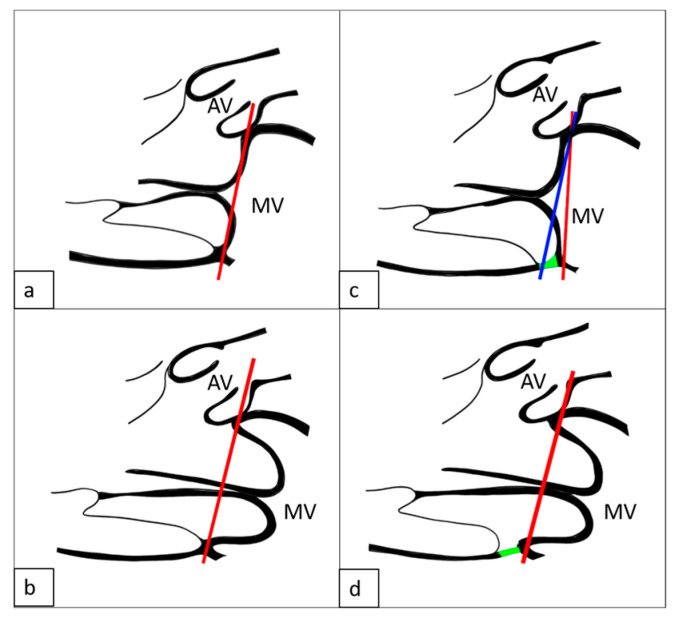
Mitral valve morphologies: (**a**) Normal reference without MAD or MVP (red line); (**b**) MVP with systolic leaflet displacement above the annular line (red line) without MAD; (**c**) MAD with superior annular displacement (blue line) and visible separation (green line) without MVP, normal Reference (red line); (**d**) combined MAD and MVP showing annular separation (green line) and systolic leaflet prolapse (red line). MAD: Mitral Annular Disjunction; MVP: Mitral Valve Prolapses; AV: Aortic Valve; MV: Mitral Valve.

Mitral annular disjunction (MAD) and mitral valve prolapse (MVP) are increasingly recognized as structurally and functionally related abnormalities within this spectrum [4,5]. MAD is characterized by a measurable separation between the hinge point of the posterior mitral leaflet and the basal left ventricular myocardium, causing atrial displacement of the annular hinge [6,7]. MVP, conversely, is defined by systolic displacement of one or both leaflets beyond the annular plane by more than 2 mm, often accompanied by leaflet thickening and redundancy [8,9] (Figure 2). Although MVP affects approximately 1–3% of the general population and is typically benign, its coexistence with MAD has been associated with arrhythmogenic potential and an increased risk of sudden cardiac death [10].

**Figure 3:**
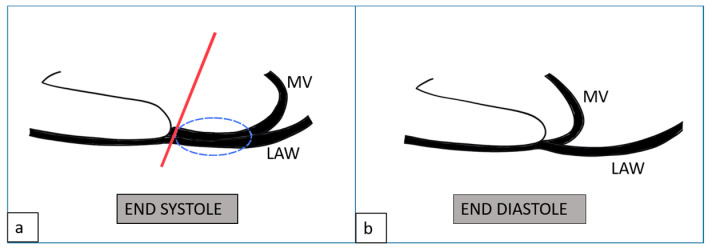
Mitral valve pathologies: (**a**) End-systolic image shows the mitral leaflet extending above the annular line (red) with atrial–ventricular separation indicated by the blue circle, seen in a patient with MVP, mimicking MAD; (**b**) in end-diastole, the apparent atrial–ventricular separation resolves, indicating pseudo-MAD with normalized annular position. MAD: Mitral Annular Disjunction; MVP: Mitral Valve Prolapses; MV: Mitral Valve.

Pseudo-mitral annular disjunction (pseudo-MAD), a commonly observed morphological variant in patients with MVP, refers to a transient systolic separation of ≥1 mm between the left atrial wall–mitral valve junction and the basal left ventricular myocardium. In contrast, true MAD is defined by a persistent atrial displacement of the mitral annulus throughout the entire cardiac cycle, typically reaching its maximum extent at end-systole [11] (Figure 3).

The recent literature has emphasized the pathophysiological interplay between MAD and MVP as part of a distinct arrhythmogenic phenotype, prompting inclusion of the “MAD-MVP complex” in the 2022 European Heart Rhythm Association (EHRA) expert consensus statement, developed in collaboration with the European Society of Cardiology (ESC) and the European Association of Cardiovascular Imaging (EACVI) [12]. This consensus underscores the importance of standardized imaging criteria and multimodal diagnostic correlation for accurate characterization and risk stratification.

While echocardiography (ECHO) remains the first-line imaging modality due to its availability, portability, and temporal resolution, its capacity for detailed anatomical evaluation is limited in subtle or complex morphologies [13]. CMR, in contrast, offers superior soft-tissue contrast, reproducibility, and three-dimensional evaluation of the mitral apparatus and surrounding myocardium [14]. Furthermore, the detection of myocardial fibrosis by means of late gadolinium enhancement (LGE) provides additional prognostic information in patients with arrhythmogenic mitral pathology [15]. However, the pediatric application of these techniques remains underexplored, with most evidence derived from adult or mixed-age cohorts.

Accordingly, the present study sought to systematically characterize mitral annular disjunction and its associated imaging findings in a pediatric population. By directly comparing echocardiographic and CMR evaluations, we aimed to determine the prevalence of MAD, explore its relationship with MVP, and assess the diagnostic performance of CMR in identifying early structural and functional changes relevant to pediatric cardiac practice.

## 2. Materials/Methods

### 2.1. Patient Selection

A total of 295 pediatric patients who underwent CMR imaging at our institution between September 2022 and March 2025 were retrospectively evaluated. Inclusion criteria comprised patients younger than 18 years of age who underwent CMR during the study period. Patients with nondiagnostic image quality or a prior history of mitral valve surgery were excluded. Demographic characteristics, clinical history, electrocardiography (ECG) results, and echocardiographic (ECHO) findings were extracted from electronic medical records. ECGs were systematically reviewed for the presence of arrhythmias or conduction abnormalities.

### 2.2. Echocardiographic Evaluation

All echocardiographic examinations were performed by a board-certified pediatric cardiologist using two-dimensional (2D), Doppler, and M-mode imaging techniques. Studies were conducted on a Vivid 7 ultrasound system (GE Vingmed Ultrasound AS, Horten, Norway) equipped with 3-MHz or 8-MHz transducers. Standard imaging planes included parasternal long- and short-axis, as well as apical two-, three-, and four-chamber views. In selected cases, nonstandard parasternal long-axis sweeps were additionally acquired to mitigate pseudo-MAD artifacts, particularly when borderline systolic separations were observed. All studies were digitally archived and systematically reviewed to ensure interstudy reproducibility and diagnostic consistency. The presence of MAD, MVP, and MR was recorded for each patient.

### 2.3. Cardiac Magnetic Resonance Imaging Protocol

CMR was performed on an Ingenia 1.5-T whole-body system (Philips Medical Systems, Best, The Netherlands) using a dedicated cardiac phased-array coil with ECG gating. All patients underwent a standardized institutional imaging protocol. Black-blood axial, sagittal, and coronal sequences were obtained in orthogonal planes for anatomical evaluation. To assess left ventricular (LV) function and wall motion, balanced steady-state free precession (bSSFP) cine images were acquired in two-chamber, four-chamber, LV outflow tract (LVOT), and contiguous short-axis planes (TR/TE = 2.7/1.2 ms, flip angle = 80°, SENSE factor = 2, voxel size = 1.8 mm × 1.8 mm × 6 mm, temporal resolution = 32.64 ms). The LVOT long-axis view was obtained by aligning the imaging plane perpendicular to the long axis of the mitral annulus and centered over the aortic outflow tract. Left ventricular ejection fraction (LVEF) was determined by manual contour tracing of endocardial and epicardial borders on end-diastolic and end-systolic frames. LGE imaging was performed 10 min after intravenous administration of gadobutrol (0.1 mmol/kg) in short-axis, two-chamber, and four-chamber planes to assess myocardial fibrosis.

### 2.4. Image Analysis

**Figure 4:**
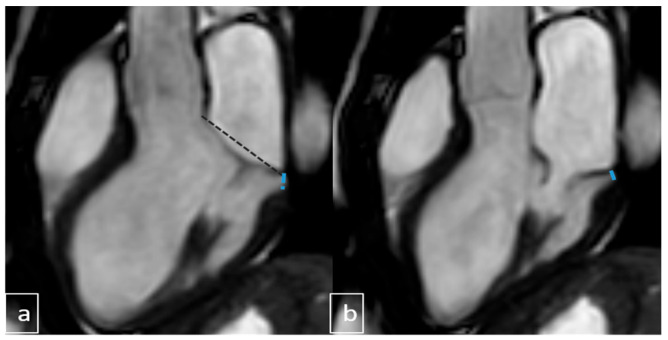
LVOT images: (**a**) end-systolic and (**b**) end-diastolic views. In cases with negative MVP but positive MAD, leaflets do not extend beyond the posterior hinge point. True MAD shows annular separation (dotted line: mitral annular diameter, blue lines: mitral annular separation area) from ventricular myocardium in both phases. LVOT: Left Ventricular Outflow Tract; MAD: Mitral Annular Disjunction.

**Figure 5:**
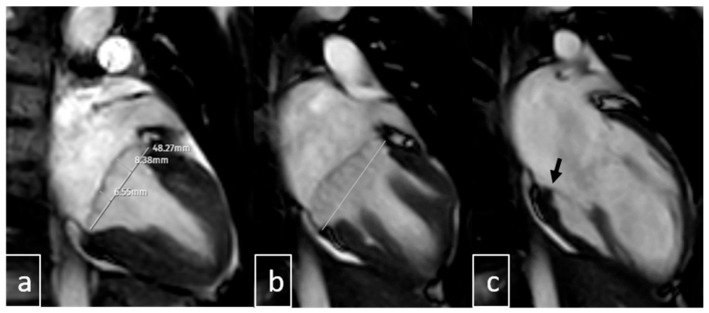
Two chamber systolic images (**a**,**b**) demonstrate anterior (8.3 mm) and posterior (6.5 mm) mitral leaflet displacement above the annular plane (white line), consistent with MVP. Diastolic imaging (**c**) shows a normally positioned annulus (black arrow) without MAD. MVP: Mitral Valve Prolapse, MAD: Mitral Annular Disjunction.

Image interpretation was performed by two pediatric radiologists, each with more than 10 years of experience in cardiac MRI (Ş.Y., B.U.). Both readers were blinded to all clinical, echocardiographic, and ECG data. nA comprehensive evaluation of mitral valve morphology was conducted, including assessment for features of connective tissue dysplasia such as annular dilatation, leaflet redundancy, chordal elongation, and overall integrity of the subvalvular apparatus. The following parameters were systematically recorded: presence of MAD, pseudo-MAD, MVP, MR, curling motion of the inferolateral LV wall, and additional cardiac or extracardiac abnormalities. Mitral annular diameters were measured during both systole and diastole. MAD was primarily assessed on three-chamber LVOT cine views at the insertion of the posterior mitral leaflet into the LV wall (Figure 4 and Figure 5). In positive cases, annular separation was further evaluated on two-chamber views to determine anterior and posterior wall involvement. True MAD was defined by the persistence of annular separation throughout the cardiac cycle, visible during both systole and diastole. Interobserver discrepancies were resolved by consensus following joint image review.

**Figure 6:**
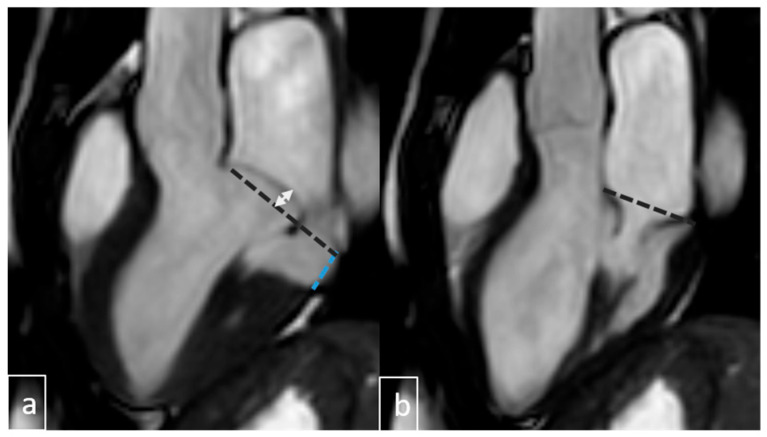
Pseudo-MAD with MVP. (**a**) At the end -sistol anterior leaflet displacement above (white double arrow) annular line (black dotted line) with annular seperation (blue dotted line). (**b**) At end-diastole, the annulus is normally positioned along the annular line (black dotted line), with no evidence of annular separation.

**Figure 7:**
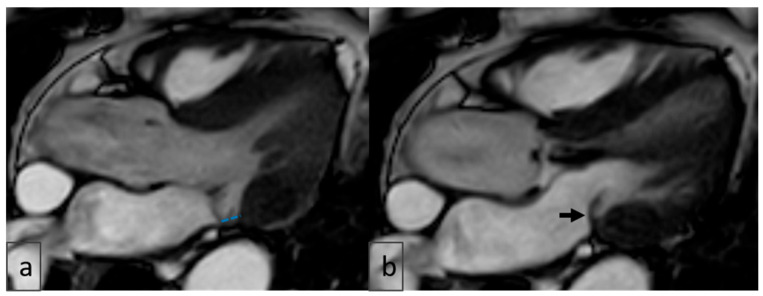
LVOT views: (**a**) End-systolic imaging shows a pseudo-MAD-like appearance (blue dotted line), raising suspicion for mitral annular disjunction; (**b**) end-diastolic imaging of the same patient does not reproduce this finding (black arrow), confirming pseudo-MAD rather than true anatomical disjunction. LVOT: Left Ventricular Outflow Tract; MAD: Mitral Annular Disjunction.

Pseudo-MAD is defined when the posterior leaflet bulges only in systole, whereas true MAD involves displacement during both diastole and systole (Figure 6 and Figure 7).

At the beginning of the study, attempts were made to measure leaflet length and thickness to assess for myxomatous degeneration; however, due to suboptimal image quality in several patients, these measurements were excluded from the final analysis.

## 3. Results

### 3.1. Study Population

A total of 237 pediatric patients were included in the final analysis for diagnostic evaluation, and 58 patients with inadequate image quality and a history of mitral valve surgery were excluded.

The study cohort was predominantly male (141/237; 59%), with a mean age of 14 ± 3 years. Body surface area (BSA) ranged from 0.83 to 2.1 m^2^. Palpitations represented the most common presenting symptom (*n* = 40). Table 1 summarizes the baseline demographic and clinical characteristics.

### 3.2. Echocardiographic Findings

Echocardiographic assessment revealed several concomitant structural abnormalities, including left ventricular noncompaction, increased trabecular density, right ventricular hypoplasia, and valvular anomalies affecting the tricuspid, pulmonary, and aortic valves. Additionally, congenital cardiac defects such as ventricular septal defect (VSD), atrial septal defect (ASD), and patent ductus arteriosus (PDA) were identified. The prevalence of mitral valve prolapses (MVP), mitral annular disjunction (MAD), and mitral regurgitation (MR) detected by echocardiography is summarized in Table 2.

### 3.3. Agreement Between Echocardiography and Cardiac MRI for MAD

MAD was identified in 55 patients (23.2%) by means of CMR and in 22 patients (9.3%) by echocardiography. Of the MRI-positive cases, 18 (32.7%) were concordantly detected by echocardiography, whereas 37 patients (67.3%) demonstrated no echocardiographic evidence of MAD. Conversely, 5 patients (2.1%) were classified as MAD-positive on echocardiography but negative on CMR. The overall inter-modality agreement was moderate (Cohen’s κ = 0.38; *p* < 0.001) (Table 3).

### 3.4. Agreement Between Echocardiography and Cardiac MRI for MVP

MVP was identified in 81 patients (34.2%) by means of CMR and in 53 patients (22.4%) by echocardiography. Among MRI-positive cases, 46 (56.8%) were confirmed by echocardiography, while 35 patients (43.2%) had no echocardiographic findings. Conversely, 7 patients (3.0%) demonstrated MVP on echocardiography but not on CMR. Agreement was moderate-to-substantial (Cohen’s κ = 0.57; *p* < 0.001) (Table 3).

### 3.5. Agreement Between Echocardiography and Cardiac MRI for MR

Mitral regurgitation was detected in 36 patients (15.2%) by means of CMR and in 74 patients (31.2%) by echocardiography. Of the MRI-positive cases, 23 (63.9%) were confirmed by echocardiography, while 13 (36.1%) showed no echocardiographic evidence. Conversely, 56 patients (23.6%) demonstrated MR on echocardiography but not on CMR. Inter-modality agreement was fair (Cohen’s κ = 0.24; *p* = 0.001) (Table 3).

### 3.6. Cardiac MRI Findings in MAD

The overall prevalence of MAD on CMR was 23.2% (55/237 patients), with a median disjunction length of 5 mm (IQR, 3–9 mm). The mean length of inferolateral disjunction, regarded as clinically significant, was 6.3 mm. In 17 patients (31%) with MAD, CMR was performed specifically to evaluate a potential arrhythmogenic substrate, whereas the remaining 38 (69%) underwent imaging for other clinical indications.

### 3.7. Association of MAD with Arrhythmia

**Figure 8:**
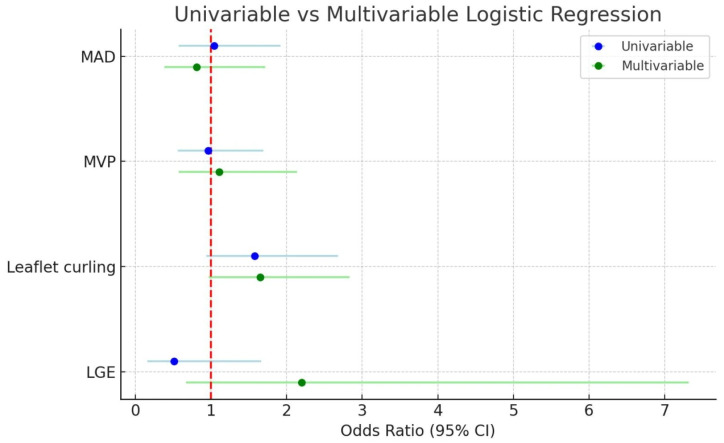
Univariable and multivariable logistic regression analysis of predictors of arrhythmia. The red dotted vertical line indicates the reference value (OR = 1.0). MAD: Mitral annular disjunction, MVP: Mitral valve prolapses, LGE: Late gadolinium enhancement.

In the univariable logistic regression analysis, none of the examined variables were significantly associated with the presence of arrhythmia. Late gadolinium enhancement (LGE) showed a non-significant trend (OR: 1.95, 95% CI: 0.60–6.34, *p* = 0.266), while leaflet curling also demonstrated a borderline association (OR: 1.58, 95% CI: 0.94–2.68, *p* = 0.087). Mitral valve prolapse (MVP) (OR: 1.03, 95% CI: 0.60–1.79, *p* = 0.894) and mitral annular disjunction (MAD) (OR: 0.96, 95% CI: 0.52–1.76, *p* = 0.891) were not significant predictors of arrhythmia in the unadjusted model. In the multivariable analysis, after adjustment for LGE, leaflet curling, MVP, and MAD, no variable reached statistical significance. LGE (OR: 2.20, 95% CI: 0.67–7.32, *p* = 0.195) and leaflet curling (OR: 1.65, 95% CI: 0.97–2.83, *p* = 0.066) again showed non-significant trends toward association, whereas MVP (OR: 1.11, 95% CI: 0.57–2.14, *p* = 0.762) and MAD (OR: 0.81, 95% CI: 0.38–1.72, *p* = 0.584) did not demonstrate meaningful associations with arrhythmia (Table 4). Univariate and Multivariate Forest Plots of Logistic Regression Results are shown in Figure 8.

**Table 4:** Disjunction Univariable and multivariable logistic regression analysis was performed to identify predictors of MAD.

	Univariable Logistic Regression Analysis		Multivariable Logistic Regression Analysis ^a^	
Variable	OR (95% CI) for Mitral Annular Disjunction MAD Presence	*p* Value	OR (95% CI) for Mitral Annular Disjunction MAD Presence	*p* Value
MVP	18.25 (8.39–39.66)	<0.001	20.09 (8.39–48.07)	<0.001
MR	2.88 (1.36–6.07)	0.006	1.67 (0.66–4.26)	0.282
Congenital cardiac disease	0.73 (0.26–2.02)	0.541	0.41 (0.12–1.42)	0.159
LVTA	0.45 (0.18–1.13)	0.088	0.44 (0.15–1.31)	0.14
Age (years)	1.09 (0.99–1.22)	0.086	1.08 (0.94–1.24)	0.283
Leaflet curling	1.96 (1.03–3.72)	0.040	1.9 (0.86–4.22)	0.115
LGE	1.70 (0.49–5.90)	0.399	3.68 (0.66–20.48)	0.136
Female sex	1.73 (0.95–3.19)	0.075	0.81 (0.36–1.79)	0.60

^a^ Adjusted for LGE, Leaflet Curling, MVP, MAD, OR = odds ratio; CI = confidence interval, LGE = late gadolinium enhancement, MVP: Mitral valve prolapses, MAD: Mitral Anuler Disjunction.

### 3.8. MAD and Its Association with Other Cardiac Conditions

Univariable logistic regression analysis demonstrated that mitral valve prolapse (MVP) was strongly associated with the presence of mitral annular disjunction (MAD) (OR: 18.25, 95% CI: 8.39–39.66, *p* < 0.001). Mitral regurgitation (MR) (OR: 2.88, 95% CI: 1.36–6.07, *p* = 0.006) and leaflet curling (OR: 1.96, 95% CI: 1.03–3.72, *p* = 0.04) were also significant predictors of MAD in the univariable model. Other variables, including congenital cardiac disease, left ventricular trabeculation (LVTA), age, late gadolinium enhancement (LGE), and female sex, were not significantly associated with MAD. In the multivariable logistic regression model, after adjustment for MVP, MR, congenital cardiac disease, LVTA, age, leaflet curling, LGE, and sex, MVP remained the only independent predictor of MAD (OR: 20.09, 95% CI: 8.39–48.07, *p* < 0.001). MR, congenital cardiac disease, LVTA, age, leaflet curling, LGE, and female sex were not independently associated with MAD in the adjusted analysis (*p* > 0.05 for all) (Table 5).

**Table 5:** Univariable and multivariable logistic regression analysis was performed to identify predictors of MAD.

	Univariable Logistic Regression Analysis		Multivariable Logistic Regression Analysis ^a^	
Variable	OR (95% CI) for Mitral Annular Disjunction MAD Presence	*p* Value	OR (95% CI) for Mitral Annular Disjunction MAD Presence	*p* Value
MVP	18.25 (8.39–39.66)	<0.001	20.09 (8.39–48.07)	<0.001
MR	2.88 (1.36–6.07)	0.006	1.67 (0.66–4.26)	0.282
Congenital cardiac disease	0.73 (0.26–2.02)	0.541	0.41 (0.12–1.42)	0.159
LVTA	0.45 (0.18–1.13)	0.088	0.44 (0.15–1.31)	0.14
Age (years)	1.09 (0.99–1.22)	0.086	1.08 (0.94–1.24)	0.283
Leaflet curling	1.96 (1.03–3.72)	0.040	1.9 (0.86–4.22)	0.115
LGE	1.70 (0.49–5.90)	0.399	3.68 (0.66–20.48)	0.136
Female sex	1.73 (0.95–3.19)	0.075	0.81 (0.36–1.79)	0.60

^a^ Adjusted for MVP, MR, Congenital cardiac disease, LVTA, Age, Leaflet Curling, LGE, Sex, OR = odds ratio, CI = confidence interval, MVP: Mitral valve prolapses, MR: mitral regurgitation, LVTA = left ventricular trabeculation, LGE = late gadolinium enhancement.

### 3.9. The Assessment of Interobserver Agreement

The assessment of interobserver agreement demonstrated excellent reproducibility across all evaluated parameters. Mitral regurgitation showed a kappa value of 0.79 with an agreement rate of 94.1%. Mitral valve prolapses yielded the highest consistency, with a kappa value of 0.85 and an agreement rate of 93.2%. For mitral annular disjunction, interobserver reliability was also high (κ = 0.77), with an agreement of 91.5%. These findings indicate substantial to almost perfect agreement among observers for all measurements (Table 6).

**Table 6:** Interobserver agreement and kappa values.

Variable	κ	Interobserver Agreement
MR	0.79	94.1
MVP	0.85	93.2
MAD	0.77	91.5

MR: Mitral Regurgitation, MVP: Mitral Valve Prolapses, MAD: Mitral Annular Disjunction.

### 3.10. Additional Imaging Findings

End-diastolic (ED) and end-systolic (ES) diameters at the mitral valve level were evaluated in all patients. ED and ES diameters were largely consistent in the MAD and non-MAD groups, and there were no significant differences (*p* = 0.086).

Intramyocardial late gadolinium enhancement was identified in 12 patients. LGE was localized to the left ventricular free wall in four cases with concomitant MVP and MAD, while no enhancement was observed in isolated MAD. Papillary muscle involvement was absent in all patients.

## 4. Discussion

The mitral valve has long been a subject of extensive investigation owing to its complex anatomical architecture and crucial contribution to maintaining physiological cardiac function. Mitral valve pathology represents a major clinical indication for cardiac magnetic resonance (CMR) imaging because of the modality’s unparalleled capability for detailed morphological delineation and comprehensive functional assessment [16,17].

In the present study, we systematically reviewed CMR examinations performed in a pediatric cohort to characterize mitral valve abnormalities, with particular emphasis on the identification of mitral valve prolapse and mitral annular disjunction. Their respective CMR characteristics were correlated with echocardiographic findings, while accompanying clinical, electrocardiographic, and demographic data were also analyzed to provide a holistic evaluation of this population.

Our comparative analysis between echocardiography and CMR revealed variable levels of agreement across parameters. While CMR demonstrated a lower diagnostic concordance for mitral regurgitation (MR)—likely due to the qualitative, visually based nature of MR assessment—it showed moderate agreement for MAD and moderate-to-substantial agreement for MVP.

The prevalence of MAD was markedly higher when assessed by means of CMR (23.2%) than by echocardiography (9.3%), with only moderate inter-modality concordance. These findings suggest that echocardiography may underestimate the presence of MAD, especially in subtle cases with minimal annular displacement. Previous investigations, particularly in homogeneous populations such as pediatric patients with Marfan syndrome, have demonstrated much higher agreement between modalities [18]. Similar concordance has been described in adult cohorts under standardized imaging protocols [19].

Accurate diagnosis of MAD requires meticulous attention to potential imaging artifacts, particularly pseudo-MAD—a systolic-only apparent separation of ≥1 mm between the left atrial wall–mitral valve junction and the basal left ventricular myocardium. True MAD, by contrast, is characterized by persistent atrial displacement of the annular hinge throughout both systole and diastole, with maximal excursion typically observed at end-systole [10,20]. In our study, careful cine-phase analysis was performed to avoid misclassification, and systolic-only separations not visible in diastole were excluded from the MAD group.

The higher frequency of MAD detected by means of CMR in this cohort likely reflects the clinical selection of patients with suspected or established cardiac pathology, as healthy controls were not included. Moreover, the superior spatial resolution and multiplanar capabilities of CMR enable more accurate delineation of annular morphology compared with echocardiography, particularly in complex or borderline cases.

MVP was also identified more frequently on CMR (34.2%) than on echocardiography (22.4%), with moderate-to-strong inter-modality agreement. This underscores echocardiography’s reliability as a first-line diagnostic tool while highlighting the incremental value of CMR in precisely characterizing leaflet morphology and identifying subtle annular abnormalities [21,22].

Assessment of MR with CMR remains technically challenging due to the valve’s rapid, multidirectional motion, the presence of turbulent flow, and the inherently small, thin structure of the mitral leaflets. Limitations in both temporal and spatial resolution complicate quantitative MR evaluation. Therefore, cine imaging remains the preferred approach for visual assessment of valve motion and regurgitant severity, especially in pediatric cohorts. Turbulent regurgitant jets manifest as hypointense signal voids due to spin dephasing, facilitating qualitative estimation of MR severity [8,9,10].

In our cohort, echocardiography detected MR in 31.2% of patients, whereas CMR identified MR in 15.2%. Although these rates are not directly comparable to quantitative flow-based measurements, they reveal limited agreement between modalities. This discrepancy may reflect both methodological and population-based differences, as well as the absence of quantitative MR assessment in our study [23,24].

Late gadolinium enhancement (LGE) findings in our cohort were limited to a small subset of patients presenting with combined MAD and MVP. No cases of isolated MAD demonstrated LGE positivity, and no significant correlation between LGE and arrhythmia was observed. Previous studies have reported substantially higher LGE prevalence in adult MVP–MAD populations, particularly involving the papillary muscles and adjacent basal myocardium [5,25]. The relative absence of LGE in our pediatric cohort likely reflects both the earlier stage of disease evolution and age-related myocardial resilience, suggesting that myocardial fibrosis may represent a later manifestation of chronic mechanical stress.

This retrospective, single-center study has several limitations. Quantitative techniques such as T1 mapping and phase-contrast flow imaging were not available for all patients, and sedation or breath-hold variability may have affected image quality. The use of a referred, nonrandomized population limits generalizability, while the retrospective design precludes causal inference. Moderate inter-modality concordance likely reflects both population heterogeneity and technical variability across imaging platforms. Future studies employing standardized acquisition parameters, prospective multicenter enrollment, and longitudinal follow-up could provide more robust insights into the natural history and clinical significance of MAD in pediatric populations.

Finally, recognition of pseudo-MAD as a potential echocardiographic artifact reinforces the need for methodological refinement and multimodal confirmation. The combined use of echocardiography and CMR offers a synergistic framework for the accurate diagnosis, risk stratification, and longitudinal evaluation of pediatric patients with suspected mitral annular pathology.

## 5. Conclusions

Our study demonstrates variable degrees of concordance between echocardiography and cardiac magnetic resonance (CMR) in the evaluation of mitral valve pathology. While the detection of mitral valve prolapses (MVP) exhibited moderate-to-substantial agreement between modalities, mitral annular disjunction (MAD) was more frequently identified by means of CMR, reflecting its superior spatial resolution and anatomic delineation. Quantification of mitral regurgitation (MR) showed only fair concordance, underscoring the inherent challenges of nonquantitative assessment. Collectively, these findings highlight the complementary role of CMR to echocardiography, particularly in diagnostically equivocal cases, and emphasize its potential utility for comprehensive characterization and risk stratification of mitral valve disease in the pediatric population.

## Figures and Tables

**Table 1:** Demographic and Clinical Characteristics of the Study Population.

Variable	Value (*n* = 237)
Age (years), mean ± SD	14.13 ± 3.16
Sex, *n* (%)	
Female	96 (41%)
Male	141 (59%)
Body surface area (m^2^), mean ± SD	1.50 ± 0.28
Presenting symptoms, *n* (%)	
Palpitations	140 (59%)
Chest pain	90 (38%)
Shortness of breath	7 (3%)

SD: Standard Deviation.

**Table 2:** Echocardiographic Findings of the Study Population.

Variable	*n* (%)
MAD	22 (9.3)
MVP	53 (22.4)
MR	74 (31.2)

**Table 3:** Concordance between ECHO and CMR in the detection of MAD, MVP, and MR.

Variable	MAD, *n* (%)	MVP, *n* (%)	MR, *n* (%)
ECHO	22 (9.3)	53 (22.4)	74 (31.2)
CMR	55 (23.2)	81 (34.2)	36 (15.2)
Cohen’s kappa coefficient	0.38	0.57	0.24

Data are expressed as number (percentage). Inter-modality agreement was assessed using Cohen’s kappa coefficient. ECHO: Echocardiography, CMR: Cardiac Magnetic Resonance Imaging, MAD: Mitral Annular Disjunction, MVP: Mitral Valve Prolapse, and MR: Mitral Regurgitation.

## Data Availability

The datasets generated and/or analyzed during this current study are available from the corresponding author upon reasonable request as they were exported from the local PACS system and are not part of an openly available repository.

## Abbreviations

The following abbreviations are used in this manuscript:

MAD	Mitral annular disjunction
MVP	Mitral annular Prolapses
MR	Mitral Regurgitation
CMR	Cardiac Magnetic Resonance
LVOT	Left Ventricular Out Flow Tract
LGE	Late gadolinium enhancement
ECHO	Echocardiography
ECG	Electrocardiography
BMI	Body Mass Index
EF	Ejection Fraction
